# Toward Next‐Generation High‐Speed Communication and Imaging: Solution‐Processed Sb_2_(S,Se)_3_/CdS Heterojunction for Ultrafast Self‐Powered Photodetector

**DOI:** 10.1002/advs.202523110

**Published:** 2026-03-15

**Authors:** Xuhua Xiao, Yichao Wang, Jingxuan Bian, Hailong Wang, Yongqiang Wang, Jinpeng Xu, Junwei Chen

**Affiliations:** ^1^ School of Electronic Engineering, North China University of Water Resources and Electric Power Zhengzhou P. R. China; ^2^ School of Microelectronics, Hefei University of Technology Hefei P. R. China; ^3^ Institute of Physics, Henan Academy of Sciences Zhengzhou P. R. China; ^4^ The Affiliated Cancer Hospital of Zhengzhou University & Henan Cancer Hospital Zhengzhou P. R. China

**Keywords:** broadband responsivity, light communication and imaging, Sb2(S,Se)3, wide‐temperature photodetection

## Abstract

The pursuit of multifunctional photodetectors (PDs) that combine broadband responsivity, high specific detectivity under low‐light conditions, and robust operation across extreme temperatures is a cornerstone for advancing next‐generation optical communication and imaging systems. Herein, we report a self‐powered PD FTO/CdS/Sb_2_(S,Se)_3_/Au via a low‐cost solution‐processed method. At zero bias, the Sb_2_(S,Se)_3_‐based PD exhibits a responsivity of 0.6 A/W, a specific detectivity of 7.68 × 10^12^ Jones, ultrafast response/recovery times of 1.13/1.90 µs, and a 3 dB bandwidth of 175 kHz under 785 nm illumination (4.09 µW/cm^2^). Most strikingly, the unencapsulated device exhibits excellent stability from 10 to 575 K, which is rarely reported for Sb_2_(S,Se)_3_‐based PDs. The practical multifunctional applicability of this single PD is further validated by its successful integration as a high‐speed receiver in a visible light communication system and a sensing pixel for high‐resolution imaging. Compared with the reference Sb_2_(S,Se)_3_ sample, the performance enhancement of the Sb_2_(S,Se)_3_/CdS heterojunction PD arises from the built‐in electric field (*E*_bi_) that enables the effective carrier separation/extraction, and the heterojunction structure that reduces the interface/bulk trap densities of the Sb_2_(S,Se)_3_ film. This work opens a promising avenue in near‐infrared imaging, light communication and extreme‐environment sensing.

## Graphical abstract

A high‐quality solution‐processed Sb_2_(S,Se)_3_/CdS heterojunction is designed to overcome the intrinsic limitations of conventional photodetectors. The fabricated device achieves an excellent self‐powered performance, including a high responsivity of 0.6 A W^−1^, an ultrahigh specific detectivity of 7.68 × 10^12^ Jones, and a wide 3db bandwidth of 175 kHz. Notably, it exhibits record‐wide operational stability across an extreme temperature range of 10‐575 K, enabling high‐speed visible light communication in the Internet of Things and high‐resolution imaging for aerospace and medical diagnostics.

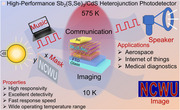

## 1 Introduction

The rapid development of advanced technologies such as 5G/6G communication, deep‐space exploration, and high‐resolution medical imaging has placed enormous demands on high‐performance photodetectors (PDs), which are the core sensing units in modern optical systems [1, 2, 3, 4, 5, 6, 7, 8]. Next‐generation applications impose a complex set of requirements: broadband spectral response to capture multi‐wavelength signals covering 400–760 nm for visible light communication (VLC) and the near‐infrared (NIR) region for imaging [9, 10, 11], high specific detectivity (D*) under low photon flux for night vision and secure communications [4, 12], resilience against wide temperature extremes encountered in aerospace and industrial environments [13, 14, 15], and intrinsic high‐speed response to enable high‐frequency optical data links. The simultaneous fulfillment of all these criteria within a single device architecture remains a significant challenge, driving the search for new material platforms and integration strategies. Beyond these performance demands, there is a pressing need for scalable and cost‐effective fabrication methods compatible with industrial production and flexible electronics [16].

Current commercial and emerging PD technologies present critical limitations that hinder their application in the multifaceted roles. For instance, while silicon‐based detectors benefit from mature processes and low cost, their operational temperature range is rather narrow. Their performance degrade severely at extreme temperatures due to impurity trap states at low temperature and drastically increased dark current at high temperatures [17, 18, 19]. III‐V semiconductors (e.g., InGaAs) exhibit excellent near‐infrared performance but are constrained by high fabrication costs, toxicity, and poor scalability [20, 21, 22]. Metal sulfide or oxide‐based devices (e.g., CdS, ZnO) often suffer from rapid carrier recombination and high interface defect densities, which result in slow response times and limited D*, failing to meet the demands of advanced optoelectronics [23, 24, 25]. Collectively, these limitations confirm an urgent need for a new PD that combines broadband response, high detectivity, operational robustness, and fabrication scalability.

Emerging as a new type of chalcogenide semiconductor, antimony selenosulfide (Sb_2_(S,Se)_3_) provides an ideal material platform for addressing the aforementioned challenges, thanks to its unique crystal structure and electronic properties [16, 26, 27, 28, 29]. Its bandgap can be tuned via the Se/S ratio (1.1–1.7 eV), which matches the broadband response range from visible light to NIR. Meanwhile, Sb_2_(S,Se)_3_ exhibits a high absorption coefficient (>10^5^ cm^−1^), low defect state density, and environmental friendliness (free of heavy metals like Pb and Hg), which endows it with great potential for low‐cost, high‐performance optoelectronic devices [27, 30, 31]. Recently, researchers have attempted to employ Sb‐based materials in PDs. However, single Sb‐based devices generally suffer from limited response speeds (e.g., typically >100 µs) due to its low carrier separation/extraction efficiency and high interfacial recombination, while the heterojunction PDs show a narrow operating temperature range and poor stability [32, 33, 34].

Previous studies have demonstrated that constructing and modifying heterojunctions can generate a built‐in electric field (*E*_bi_) at the interface. This field promotes efficient carrier separation and extraction, which drives heterostructure widespread application in solar cells, photocatalytic hydrogen production, and photodetection [9, 35, 36, 37]. Specifically, combining a suitable n‐type semiconductor with Sb_2_(S,Se)_3_ can generate an *E*_bi_ that dramatically enhances carrier separation and suppresses charge recombination, while also blocking reverse carrier tunneling to minimize leakage current [5, 38, 39, 40]. CdS, an n‐type semiconductor (∼2.4 eV), is an ideal electron transport layer (ETL) material compared to wide bandgap semiconductors (ZnO,TiO_2_). It can form a well‐matched type‐II heterojunction with Sb_2_(S,Se)_3_, facilitating efficient charge separation/extraction, while its excellent chemical stability and film‐forming properties are beneficial for device integration [41, 42]. Our previous investigations have demonstrated the application of the high‐quality Sb_2_(S,Se)_3_/CdS heterojunction in solar cells [16, 27, 28, 29, 30, 31]. However, the full potential of the Sb_2_(S,Se)_3_/CdS heterojunctions remains largely unexplored, particularly for ultrafast response, extreme temperature stability, and direct integration into functional systems such as VLC and high‐resolution imaging. This presents significant challenges but promising prospects.

To directly address these critical issues, the well‐formed Sb_2_(S,Se)_3_/CdS heterojunctions were fabricated via a precision‐controlled hydrothermal process. This approach can increase the grain size and reduce the trap density of Sb_2_(S,Se)_3_ film, while enhancing the *E*_bi_ of the heterojunction. Consequently, the Sb_2_(S,Se)_3_/CdS heterojunction PDs exhibit ultrafast response, excellent broadband detectivity, and remarkable temperature stability in a self‐powered mode, arising from favorable heterointerface contact and efficient charge transport. At zero bias, the vertically structured PD FTO/CdS/Sb_2_(S,Se)_3_/Au exhibits a high responsivity (*R*) of 0.6 A/W, a specific detectivity of 7.68 × 10^12^ Jones, and fast response speeds of 1.81/2.82 µs (22 kHz), 1.13/1.90 µs (200 kHz) under 4.09 µW/cm^2^, 785 nm illumination in ambient conditions. More strikingly, the unencapsulated Sb_2_(S,Se)_3_‐based PD maintains full functionality from 10 to 575 K, far exceeding the operating temperature limits of conventional silicon‐based PDs. Such a systematic evaluation of wide‐temperature operational stability has rarely been reported for Sb_2_(S,Se)_3_‐based PDs. The practical multifunctional superiority of this single PD is validated by its integration into a visible‐light communication system as a high‐speed receiver and a high‐resolution imaging pixel. This work sets a new benchmark for low‐cost, self‐powered PDs and paves the way for the potential application in advanced optical communication and imaging, such as medical NIR imaging, aerospace extreme‐temperature detection, and security multispectral surveillance.

## 2 Results and Discussion

**FIGURE 1:**
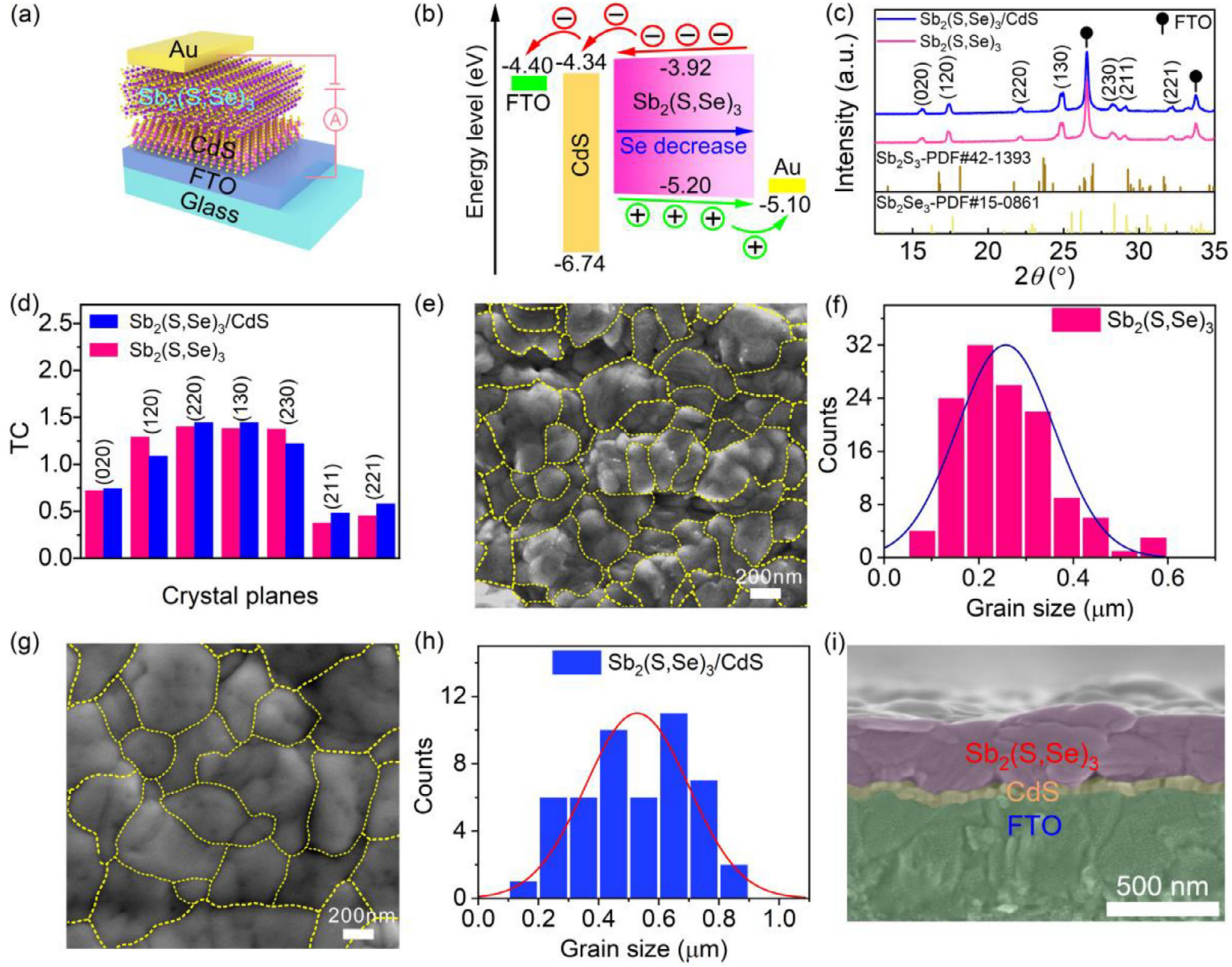
(a) Device architecture of the Sb_2_(S,Se)_3_‐based PD. (b) Energy band diagram of Sb_2_(S,Se)_3_/CdS heterojunction PD. (c) XRD patterns of the Sb_2_(S,Se)_3_/CdS and Sb_2_(S,Se)_3_ films. (d) TC values of Sb_2_(S,Se)_3_ and Sb_2_(S,Se)_3_/CdS samples. (e,g) Top‐view SEM images and (f,h) crystal size distributions of the reference Sb_2_(S,Se)_3_ and Sb_2_(S,Se)_3_/CdS films. (i) Cross‐sectional SEM image of the Sb_2_(S,Se)_3_/CdS heterojunction.

Figure 1a depicts the device structure: FTO/CdS/Sb_2_(S,Se)_3_/Au. This heterostructure can enhance the performance of Sb_2_(S,Se)_3_‐based PDs compared to conventional photoconductive designs due to the efficient separation of photogenerated electron‐hole pairs driven by the *E*_bi_ at the Sb_2_(S,Se)_3_/CdS interface [43, 44, 45]. Electrical contacts are formed using Au on Sb_2_(S,Se)_3_ film as the anode and the exposed FTO edge as the cathode. The corresponding energy band diagram in Figure 1b demonstrates a favorable band alignment with a graded bandgap. Such a gradual variation in energy levels effectively promotes efficient charge separation and transport, which is responsible for the excellent self‐powered photodetection performance [46]. Structural characterization of the Sb_2_(S,Se)_3_ film was performed using X‐ray diffraction (XRD). Nearly all diffraction peaks align well with the standard orthorhombic Sb_2_S_3_ (JCPDS No. 42–1393) and Sb_2_Se_3_ (JCPDS No. 15–0861) reference patterns (Figure 1c), which confirms the high phase purity and crystalline quality of the synthesized films. Distinct diffraction peaks are observed at 17.28°, 24.76°, 28.14°, 29.03°, and 32.08°, which correspond to the (120), (130), (230), (211), and (221) crystal planes of Sb_2_(S,Se)_3_, respectively, exhibiting a typical orthorhombic structure with a *Pbnm* space group [47]. Notably, the diffraction peaks of Sb_2_(S,Se)_3_ lie between those of pure Sb_2_S_3_ and Sb_2_Se_3_, which indicates the successful incorporation of Se atoms into the crystal lattice (Figure S1) [27, 28, 29]. Meanwhile, the texture coefficient (TC) of Sb_2_(S,Se)_3_/CdS and Sb_2_(S,Se)_3_ provides supplementary evidence that the introduction of CdS promotes the weak preferential orientation of Sb_2_(S,Se)_3_ along the c‐axis direction ([*hkl*, *l* ≠ 0], Figure 1d). The Sb_2_(S,Se)_3_/CdS heterojunction film shows higher diffraction peak intensities compared to the reference sample, which suggests improved crystallinity due to the inducing effect of CdS crystals [30]. Raman spectroscopy reveals that the Sb_2_(S,Se)_3_ film exhibits characteristic peaks at ca.150, 192, and 295 cm^−1^, which correspond to the stretching vibration of Sb‐Sb, Sb‐S(Se) bonds. The dominant Sb‐S(Se) peaks at ∼192 and 295 cm^−1^ show reduced FWHM values in the Sb_2_(S,Se)_3_/CdS film compared with the reference Sb_2_(S,Se)_3_ film, which indicates higher crystalline quality, less lattice disorder, and fewer defects in the Sb_2_(S,Se)_3_/CdS film (Figure S2; Table S1) [9, 33].

The surface morphology of the Sb_2_(S,Se)_3_ films was examined by top‐view scanning electron microscopy (SEM). Compared to the reference Sb_2_(S,Se)_3_ sample with an average grain size of 257 nm, the Sb_2_(S,Se)_3_/CdS film displays a markedly smoother surface with a statistical crystal size of 525 nm, which suggests a significantly reduced density of Sb_2_(S,Se)_3_ grain boundaries (Figure 1e–h). These optimized morphological characteristics are critical for suppressing non‐radiative recombination losses and promoting efficient charge transport and interfacial separation, factors that directly contribute to the enhancement of Sb_2_(S,Se)_3_‐based PD performance [27]. A cross‐sectional SEM image of the Sb_2_(S,Se)_3_/CdS heterojunction reveals a well‐defined layered architecture, with a Sb_2_(S,Se)_3_ thickness of ca. 300 nm (Figure 1i). This well‐structured interface facilitates the separation and collection of photogenerated carriers, which accounts for the excellent detection performance and temperature stability of the Sb_2_(S,Se)_3_‐based PDs.

The Sb_2_(S,Se)_3_/CdS sample exhibits broadband absorption from the ultraviolet (UV) to NIR region (300–1100 nm) and a direct bandgap (*E*_g_) of ca. 1.28 eV according to Tauc plots, similar to the reference Sb_2_(S,Se)_3_ film (Figure S3) [48]. The above broadband absorption characteristic is suitable for photodetection applications that require multi‐wavelength signal capture, such as VLC and NIR imaging. The Sb_2_(S,Se)_3_/CdS film shows a reduced photoluminescence PL intensity compared to the reference Sb_2_(S,Se)_3_ film (Figure S4a), which indicates the efficient charge transfer at the Sb_2_(S,Se)_3_/CdS heterojunction interface. Furthermore, the time‐resolved photoluminescence (TRPL) spectroscopy reveals that the Sb_2_(S,Se)_3_/CdS heterojunction has an average carrier lifetime of 16 ns (biexponential fitting), much shorter than the 82 ns of the reference film (Figure S4b; Table S2). This reduction in lifetime further evidences the rapid charge separation and transfer, which can effectively minimize non‐radiative recombination losses. Notably, the nanosecond‐scale lifetime ensures sufficient time for efficient charge collection, a key factor contributing to the excellent performance of Sb_2_(S,Se)_3_/CdS heterojunction PDs and photovoltaic devices [31].

**FIGURE 2:**
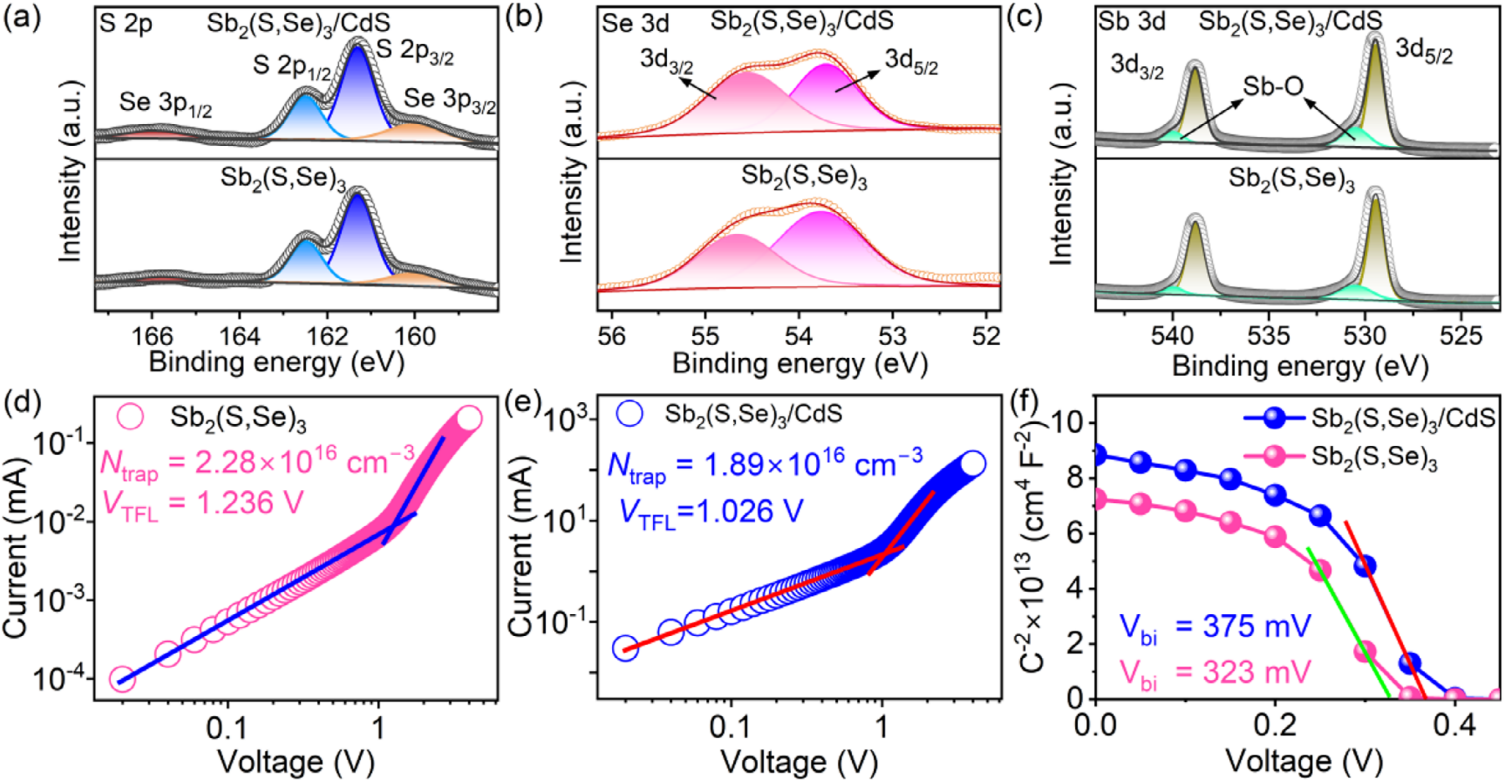
High‐resolution XPS spectra of (a) S 2p, (b) Se 3d and (c) Sb 3d for Sb_2(_S,Se)_3_ and Sb_2_(S,Se)_3_/CdS films. Electron‐only current–voltage (*I–V*) curves for the SCLC tests of (d) Sb_2(_S,Se)_3_ and (e) Sb_2_(S,Se)_3_/CdS samples. (f) *C* ^−2^‐*V* curves of Sb_2(_S,Se)_3_ and Sb_2_(S,Se)_3_/CdS devices.

X‐ray photoelectron spectroscopy (XPS) characterization was conducted to study the chemical composition and surface electronic states of both Sb_2_(S,Se)_3_ and Sb_2_(S,Se)_3_/CdS samples. Both samples display characteristic S 2p signals at 161.31 eV (2p_3/2_) and 162.48 eV (2p_1/2_), which are indicative of S^2−^ anion existence in the Sb_2_(S,Se)_3_ films (Figure 2a). Further deconvolution of the S 2p core‐level spectra reveals slightly enhanced peaks at Se 3p_3/2_ peak at 160.03 eV and Se 3p_1/2_ peak at 165.78 eV, which affirms more selenium incorporation into the Sb_2_(S,Se)_3_ film with CdS substrate. Further analysis of the Se 3d region indicates that the doublet peaks remain fixed at 53.74 eV (3d_5/2_) and 54.60 eV (3d_3/2_) (Figure 2b). In addition, the Sb_2_(S,Se)_3_/CdS sample exhibits intensified Se 3d areas, which further suggests an increased Se content in the Sb_2_(S,Se)_3_ film. The Sb 3d core‐level spectra show characteristic signals at 529.43 eV (3d_5/2_) and 538.80 eV (3d_3/2_), consistent with the covalent Sb–(S,Se) bonding of the Sb_2_(S,Se)_3_ films (Figure 2c; Table S3) [27, 49].

The space charge‐limited current (SCLC) was employed with a device structure of FTO/CdS/Sb_2_(S,Se)_3_/PCBM/Au to gain deeper insight into the defect states by analyzing the dark logarithmic *I‐V* curves (Figures 2d,e). Consequently, the derived onset voltages (*V*_TFL_) for the Sb_2_(S,Se)_3_/CdS and Sb_2_(S,Se)_3_ devices are 1.026 and 1.236 V, respectively. The defect density (*N*_trap_) was then calculated using the following equation: *N*_trap_ = 2*ε_0_ ε*_r_ *V_TFL_*/*qd* ^2^, where *ε_0_* denotes vacuum permittivity, *ε_r_* is the relative permittivity of the material, *q* represents electron charge, and *d* is the thickness of the active layer. The SCLC curves show that the reference Sb_2_(S,Se)_3_ device corresponds to a trap density of 2.28 × 10^16^ cm^−3^. After CdS deposition, the *N*_trap_ value of Sb_2_(S,Se)_3_/CdS device decreases to 1.89 × 10^16^ cm^−3^. This result directly confirms that the existence of CdS layer effectively suppresses the defect density in bulk phase Sb_2_(S,Se)_3_ and at the Sb_2_(S,Se)_3_/CdS heterojunction interface, which is in accordance with previous literature [50].

To further evaluate the quality of the Sb_2_(S,Se)_3_/CdS heterojunction, capacitance–voltage (*C‐*‐*V*) curves for Sb_2_(S,Se)_3_/CdS and Sb_2_(S,Se)_3_ were measured (Figure 2f). The built‐in voltage (*V*_bi_) was then evaluated using the following equation: *C* ^−2^ = 2(*V*‐*V*_bi_)/q*ε*_r_ *ε*_0_ *N*_A_A^2^, where *C* is the space‐charge capacitance, *V* signifies the applied bias, *ε*_r_ and *ε*_0_ denote the relative permittivity and the vacuum permittivity, *N*_A_ represents the acceptor density, and *A* indicates the electrode area. Correspondingly, the calculated *V*_bi_ values are 0.375 V for the Sb_2_(S,Se)_3_/CdS heterojunction and 0.323 V for reference Sb_2_(S,Se)_3_ device, respectively. Given the similar active layer thickness, clearly, the *E*_bi_ (*E*_bi_ = *V*_bi_/d) of CdS/Sb_2_(S,Se)_3_ heterojunction device is higher than that of the reference Sb_2_(S,Se)_3_ device, directly confirming a stronger *E*_bi_ that accelerates the photogenerated carrier transfer and extraction.

**FIGURE 3:**
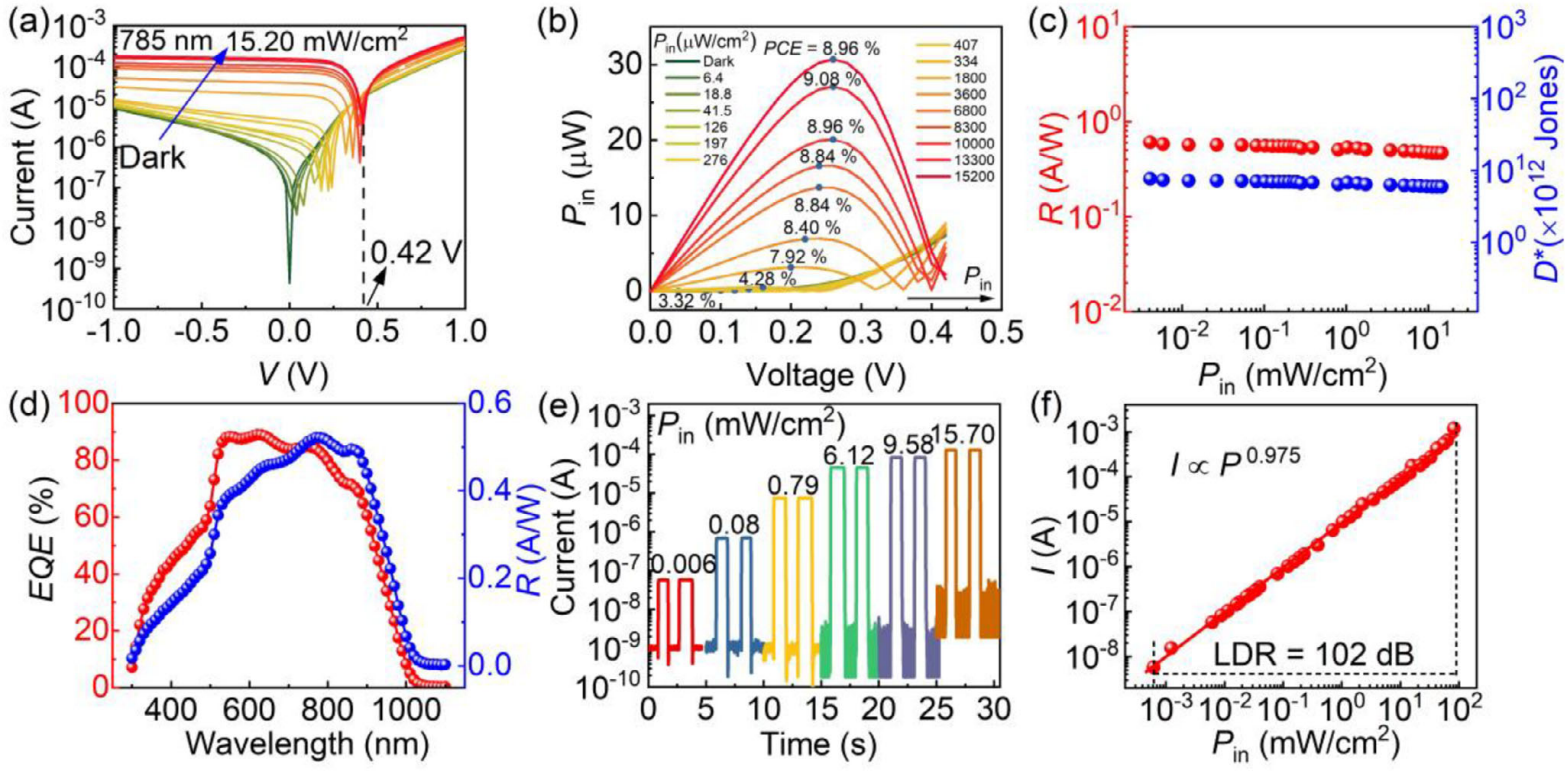
Optoelectronic characterization tested at 785 nm. (a) Current‐voltage characteristics of the PD measured under different illumination intensities. (b) PCE of Sb_2_(S,Se)_3_/CdS heterojunction PD. (c) *R*, D^*^ of the PD versus light intensity. (d) *EQE* of the Sb_2_(S,Se)_3_/CdS heterojunction PDs versus wavelength. (e) Photocurrent response tested at 0 V bias. (f) Dependence of photocurrent on light irradiation power intensity at 0 V bias.

The current–voltage (*I*–*V*) characteristics of the Sb_2_(S,Se)_3_/CdS heterojunction PD under 785 nm illumination reveal pronounced diode‐like rectification behavior and a substantial open‐circuit voltage of 0.42 V (Figure 3a), which clearly indicates the formation of an *E*_bi_ at the Sb_2_(S,Se)_3_/CdS interface and confirms its compelling self‐powered capability. Both the photocurrent and photovoltage systematically increase with incident light intensity (6.4 µW cm^−2^ to 15.7 mW cm^−2^), consistent with enhanced photogenerated carrier density. Notably, at zero bias, the device current increases by over five orders of magnitude from 9.41 × 10^−10^ A (dark) to 1.95 × 10^−4^ A (15.7 mW cm^−2^ illumination). The electrical output power (*P*_el_ = *IV*) was derived from the *I*–*V* curves, which initially increases and then decreases back to zero with increasing the bias voltage at each illumination level (Figure 3b). Power conversion efficiency (PCE) is calculated by the formula PCE = *P*_max_/*P*_in,_ where *P*_max_ is the maximum electrical output power and *P*_in_ is the incident monochromatic optical power. A maximum PCE of 9.08% is achieved at 785 nm, which surpasses most values reported Sb_2_(S,Se)_3_‐based PDs and indicates the excellent energy‐harvesting potential of the Sb_2_(S,Se)_3_/CdS heterojunction [47]. This high efficiency stems from the favorable bandgap alignment with incident photon energy and high‐quality interfacial contact characteristics.

The photodetection properties of the Sb_2_(S,Se)_3_/CdS heterojunction were quantitatively characterized by *R*, D*, external quantum efficiency (*EQE*), linear dynamic range (*LDR*) based on previous literature [57]. The responsivity, defined as *R* = (*I*_ph_‐*I*_dark_)/*P*_in_, where *I*_ph_ is the photocurrent, *I*_dark_ is the dark current and *P*_in_ is the intensity of incident light, reflects sensitivity of the device to optical signals. Under 785 nm illumination (4.09 µW cm^−2^), the detector achieves a remarkable *R* value of 0.6 A W^−1^ in self‐powered mode. The specific detectivity, a key metric for weak‐light detection, was calculated using D^*^ = *R*(*A/2eI*_dark_)^1/2^, where *A* is the device area and *e* is the charge of a single electron. As shown in Figure 3c, the device exhibits a peak D^*^ of 7.67 × 10^12^ Jones at zero bias under 785 nm illumination (4.09 µW cm^−2^), which places it among the top ranks of the recently reported high‐performance antimony chalcogenide‐based semiconductor PDs (Table 1). Consequently, the Sb_2_(S,Se)_3_/CdS heterojunction PDs achieve excellent overall photodetection performance, particularly in weak‐light scenarios. To avoid overestimating the realistic detectivity, noise equivalent power (NEP) was calculated as 5.02 × 10^−12^ W·Hz^−1/2^ according to the frequency‐dependent noise power spectra (Figure S5). Correspondingly, the specific detectivity D^*^ of the Sb_2_(S,Se)_3_/CdS heterojunction PD was calculated to be 3.12 × 10^10^ Jones. To facilitate subsequent comparisons of D^*^ at different temperatures, D^*^ = *R*(*A/2eI*_dark_)^1/2^ is utilized in the subsequent calculations. Furthermore, *EQE* defined as *EQE* = *hcR*/(*λq*), where *h* is Planck′s constant, *c* is the speed of light, *λ* is the wavelength of incident light, and *q* is the electron charge, which can reach 95% under 4.09 µW cm^−2^ illumination (Figure S6). To avoid overestimation, we measured the wavelength‐dependent spectral response (Figure 3d). Notably, the Sb_2_(S,Se)_3_/CdS heterojunction PD exhibits a broadband response from the UV to NIR region (300−1100 nm), with *EQE* exceeding 80% in the range of 500–800 nm. The calculated *R* reaches 0.57 A/W at 785 nm, which is consistent with the results shown in Figure 3c. The excellent performance of the Sb_2_(S,Se)_3_/CdS heterojunction PD stems from the low trap density within the Sb_2_(S,Se)_3_ film, coupled with efficient charge extraction and separation at the heterointerface.

**TABLE 1:** Comparison of the key parameters of our Sb_2_(S,Se)_3_‐based PDs with previous literatures.

Materials	*R* (A/W)	D^*^ (Jones)	Bias (V)	Decay time	λ (nm)	References
Sb_2_Se_3_NW_S_	8.0	No	5	<0.3 s	600	[44]
Sb_2_S_3_NW_S_	0.217	no	2	470/680 µs	638	[51]
Sb_2_S_3_NW_S_	1152	2 × 10^13^	1	37/38 ms	638	[52]
(Sb_0.44_Bi_0.56_)_2_Se_3_NW_S_	8261.4	No	5	<0.5/<0.3 s	639	[45]
Sb_2_(S_1/3_Se_2/3_)_3_NW_S_	5.48	1.23 × 10^9^	0.5	743/692 µs	638	[43]
PI/ITO/CdS/Sb_2_Se_3_/Au	0.42	2.4 × 10^11^	0	1.8/1.6 µs	625	[26]
ITO/Sb_2_Se_3_/MoO_3_/Ag	0.0173	2.25 × 10^11^	0	No	625	[53]
Sb_2_Se_3_ NF_S_/ZnO NR_S_	0.0215	5.95 × 10^13^	3	No	628	[5]
Mo/Sb_2_Se_3_/CdS(Al)/ITO/Ag	0.9	4.78 × 10^12^	0	24/75 ns	635	[54]
FTO/CdS/Sb_2_Se_3_/Au	0.47	5 × 10^12^	0	No	660	[32]
MoS_2_/MoTe_2_/MoS_2_/MoTe_2_	1.57	4.28 × 10^11^	0	30 µs	532	[55]
SnS_2_/(PEA)_2_PbI_4_	2.02	5.8 × 10^11^	1	38.5/64.2 ms	532	[56]
FTO/CdS/Sb_2_(S,Se)_3_/Au	**0.6**	**7.68 × 10^12^**	**0**	**1.81/2.82 µs**	**785**	**This work**

The dynamic photoresponse of the Sb_2_(S,Se)_3_/CdS heterojunction PD was evaluated at zero bias (Figure 3e). The device exhibits rapid and reversible switching behavior under 785 nm illumination across a wide range of incident power densities (6 µW cm^−2^ to 15.70 mW cm^−2^). A high on/off current ratio of 1.2 × 10^5^ is achieved at 9.58 mW cm^−2^, while a ratio of ∼100 is maintained even under weak light (8 µW cm^−2^), which confirms its good sensitivity in low‐signal environments. In addition, an increase in the dark current is observed under strong light exposure (15.7 mW cm^−2^). This behavior is attributed to light‐induced trap filling and transient band realignment at the Sb_2_(S,Se)_3_/CdS interface. High‐intensity illumination fills deep‐level traps within the depletion region, reducing the effective barrier height and facilitating trap‐assisted carrier injection under subsequent dark conditions [58, 59]. In contrast, the reference Sb_2_(S,Se)_3_ film (Figure S7a) shows significantly inferior performance, with a maximum *R* and D^*^ values of only ∼9.48 × 10^−3^ A W^−1^ and 1.62 × 10^9^ Jones, respectively, which are at least two orders of magnitude lower than those of the heterojunction device (Figure S7b). This dramatic enhancement originates from the well‐designed Sb_2_(S,Se)_3_/CdS interface, where a strong *E*_bi_ promotes efficient carrier separation/extraction and suppresses carrier recombination. Additionally, the superior Sb_2_(S,Se)_3_/CdS heterojunction effectively suppresses reverse tunneling currents, thereby minimizing the device leakage and establishing a new material platform for high‐performance, self‐powered photodetection (Figure 3d–f).

The photocurrent of the Sb_2_(S,Se)_3_/CdS heterojunction PDs exhibits a well‐fitted power‐law dependence on incident light intensity, following the relation *I*_p_ ∝ α*P^β^*, where *P* is the light excitation power, *α* represents a proportional constant, and *β* is an empirical parameter that reflects the degree of trap‐assisted recombination of photoexcited carriers (Figure 3f). The obtained *β* value is close to unity (0.975), a characteristic signature of an ideal quasi‐linear PD [57]. It reflects highly efficient photon‐to‐electron conversion and negligible trap‐assisted recombination at the Sb_2_(S,Se)_3_/CdS heterointerface, confirming that the high‐quality device performance is suitable for linear optical signal detection. Furthermore, the *LDR* was extracted by the *I*_ph_ *‐P* curves using the formula *LDR* = 20log (*I*_max_/*I*_min_), where *I*_max_ and *I*_min_ represent the maximum and minimum light current values, respectively. *LDR* of 102 dB was obtained in the self‐powered state, confirming the excellent photoresponse characteristic covering a wide intensity range from 0.6 µWcm^−2^ to 81.71 mWcm^−2^. Notably, the device still exhibits effective operation at an ultralow intensity of 0.6 µWcm^−2^, demonstrating excellent weak‐light detection capability owing to the high sensitivity and low noise of the Sb_2_(S,Se)_3_/CdS heterojunction PD. To systematically evaluate the spectral response of the Sb_2_(S,Se)_3_/CdS heterojunction PD, we measured the *I‐V* and *I‐T* characteristics with different monochromatic wavelength illumination (375 nm, 450 nm, 532 nm, and 1064 nm) covering the UV to NIR (Figure S8a–h). The self‐powered Sb_2_(S,Se)_3_/CdS heterojunction PD shows wavelength‐dependent performance, with optimal *R*, D^*^ and *EQE* values of 0.36 A W^−1^, 4.5 × 10^12^ Jones and 83% in the visible region (532 nm, 0.9 µW cm^−2^, Figure S8i–k), respectively. Power‐law fitting yields near‐unity *β* values for visible light (suppressed recombination) and lower values for UV/NIR (trap‐assisted recombination), which supports its suitability for VLC application (Figure S8l).

**FIGURE 4:**
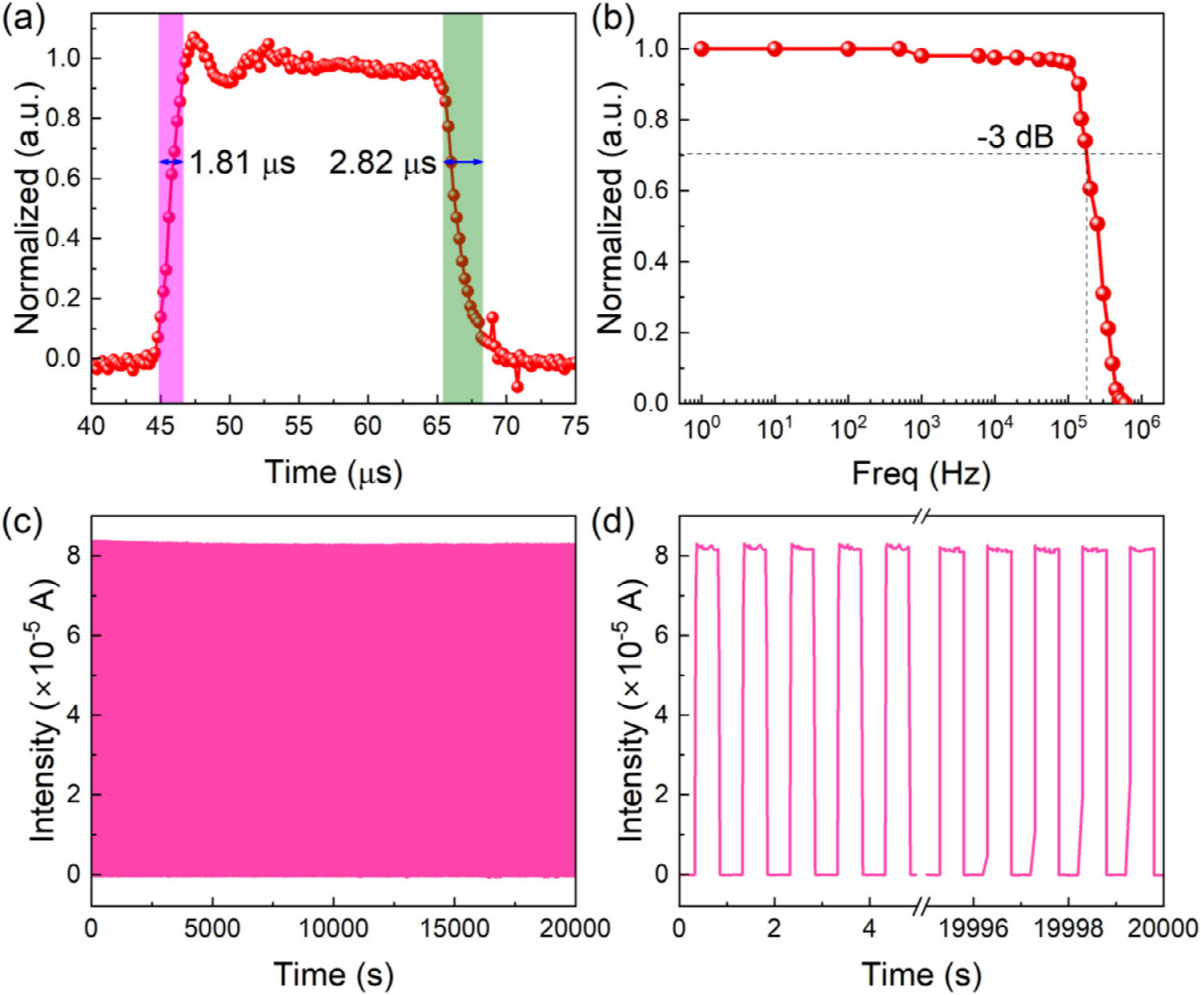
(a) Representative rising and falling edges of the photocurrent response of a single pulsed light at 22 kHz. (b) 3 dB bandwidth of Sb_2_(S,Se)_3_/CdS heterojunction PD. (c) The consecutive response measurement with 20 000 continuous cycles. (d) The first three and the last three cycles in Figure 3c.

To systematically evaluate the dynamic response speed of the Sb_2_(S,Se)_3_/CdS heterojunction PD, we used a pulsed laser to measure the transient photocurrent (Figure S9a). The device exhibits sharp and repeatable photoresponse under high‐frequency modulated illumination up to 200 kHz (Figure S9b–d), which demonstrates excellent signal‐tracking capability and operational stability. By analyzing the transient profiles at 22 kHz and 200 kHz (Figure 4a; Figure S9d), rise and fall times of 1.81/2.82 µs and 1.13/1.90 µs (10%–90%) were extracted, respectively, significantly surpassing those of most reported Sb_2_(S,Se)_3_‐based and analogous chalcogenide PDs [51, 53]. According to equation *f*_3dB_ = 0.35/*τ*_rise_, where *τ*_rise_ is the response time of the device, the intrinsic 3 dB bandwidth can be calculated to be 193 kHz [60]. However, the 3 dB bandwidth is 175 kHz according to our experimental measurements.(Figure 4b) The difference between the calculated and measured 3 dB bandwidth is possibly attributed to the RC time constant limitation of the measurement system (*f*_RC_ ≈ 152 kHz, calculated from a junction capacitance ∼1000 pF and resistance ∼1050 Ω). Such fast response speed can be mainly ascribed to the faster charge separation, transport, and extraction enabled by the type‐II band alignment at the Sb_2_(S,Se)_3_/CdS heterojunction. The built‐in electric field, verified by TRPL and *C*–*V* characterization, effectively suppresses charge recombination and facilitates rapid carrier collection.

Beyond the response speed, the operational reliability was also evaluated with prolonged cycling and ambient aging. The unencapsulated device maintains a highly reproducible response over 20 000 on/off cycles at 1 Hz (Figure 4c,d). Statistical variance analysis shows a maximum deviation of 1.22%, which confirms the robust operational stability of the device (Figure S10). Such high stability is crucial for its practical application in high‐speed optical communication systems. Meanwhile, the photocurrent of the Sb_2_(S,Se)_3_‐based PD remains nearly unchanged after one month of storage in a glovebox (Figure S11), which confirms its stability for practical application.

**FIGURE 5:**
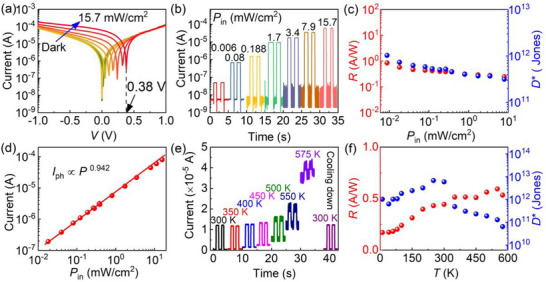
(a) The current‐voltage characteristic curves at 10 K. (b) Photocurrent response tested at 0 V bias, 10 K. (c) *R*, D^*^ versus light intensity. (d) Dependence of photocurrent on the intensity of light irradiation power at 0 V bias, 10 K. (e) Temporal photoresponse curves of the device at temperatures from 300 to 575 K. (f) *R* and D^*^ at different temperatures.

To assess the operational robustness of the unencapsulated Sb_2_(S,Se)_3_/CdS heterojunction PDs under extreme temperature conditions, we measured the *I–V* and *I–T* curves under cryogenic environment (0–300 K, a vacuum of 8 × 10^−4^ Pa) and high‐temperature (300–575 K, in ambient air with 35%–50% relative humidity) environments, respectively. All high‐temperature measurements were performed at zero bias with a fixed intensity of 0.90 mW cm^−2^ under 785 nm illumination. The open‐circuit voltage reaches 0.38 V at 10 K, which confirms the stable self‐powered operation even under cryogenic environment (Figure 5a). Additionally, the corresponding time‐resolved photoresponse at 10 K (Figure 5b) further demonstrates excellent detection capability under weak light. A comparison of dark current variations at 10 K and room temperature (Figure 3e) reveals a notably smaller fluctuation at 10 K, indicating significant suppression of both thermal excitation and defect‐assisted processes [59, 61]. The variation trends of *R* and *D* ^*^ with incident power (Figure 5c) are similar to those at room temperature, while the power‐law exponent *β* (0.942) is close to unity, indicating that carrier recombination is highly suppressed at low temperature. Additional validation under 532 nm illumination (Figure S12a,b) reveals analogous performance trends, which indicates broadband spectral operability in cryogenic environments. Notably, under ultralow light intensity (0.9 µW cm^−2^), the device maintains high responsivity of ∼0.12 A W^−1^ and detectivity of ∼3.58 × 10^11^ Jones (Figure S12c), which demonstrates its suitability for weak‐signal detection under extreme temperature conditions.

Building on the favorable cryogenic performance, we further investigated the high‐temperature operational stability of the unencapsulated Sb_2_(S,Se)_3_/CdS heterojunction PDs. As illustrated in Figure 5e, both the photocurrent and dark current increase with rising operating temperature due to the thermal excitation of charge carriers. Consequently, the dark current grows more significantly, which results in a gradual reduction in the on/off current ratio. The evolution of *R* and *D* ^*^ with temperature is summarized in Figure 5f. *R* increases moderately with temperature, while *D* ^*^ begins to decrease above 350 K due to the increase in dark current caused by the rapid temperature increase. Remarkably, even at 575 K, the device retains a detectivity of 6.42 × 10^10^ Jones. Importantly, the photocurrent and dark current variations are reversible upon cooling to room temperature, which demonstrates excellent thermal recoverability. Raman spectroscopy was performed to evaluate the structural characteristics of the Sb_2_(S,Se)_3_/CdS heterojunction after high‐temperature testing, as shown in Figure S13. The main characteristic Raman peaks of Sb_2_(S,Se)_3_ remain almost stable, which further confirms the excellent structural stability of the material under high‐temperature. These results collectively validate the robust and repeatable optoelectronic response of the Sb_2_(S,Se)_3_/CdS heterojunction across an ultra‐broad temperature range of 10–575 K. Such consistent performance under extreme temperatures and weak illumination underscores the potential of this technology for demanding applications in quantum computing, aerospace systems, and high‐resolution medical imaging.

**FIGURE 6:**
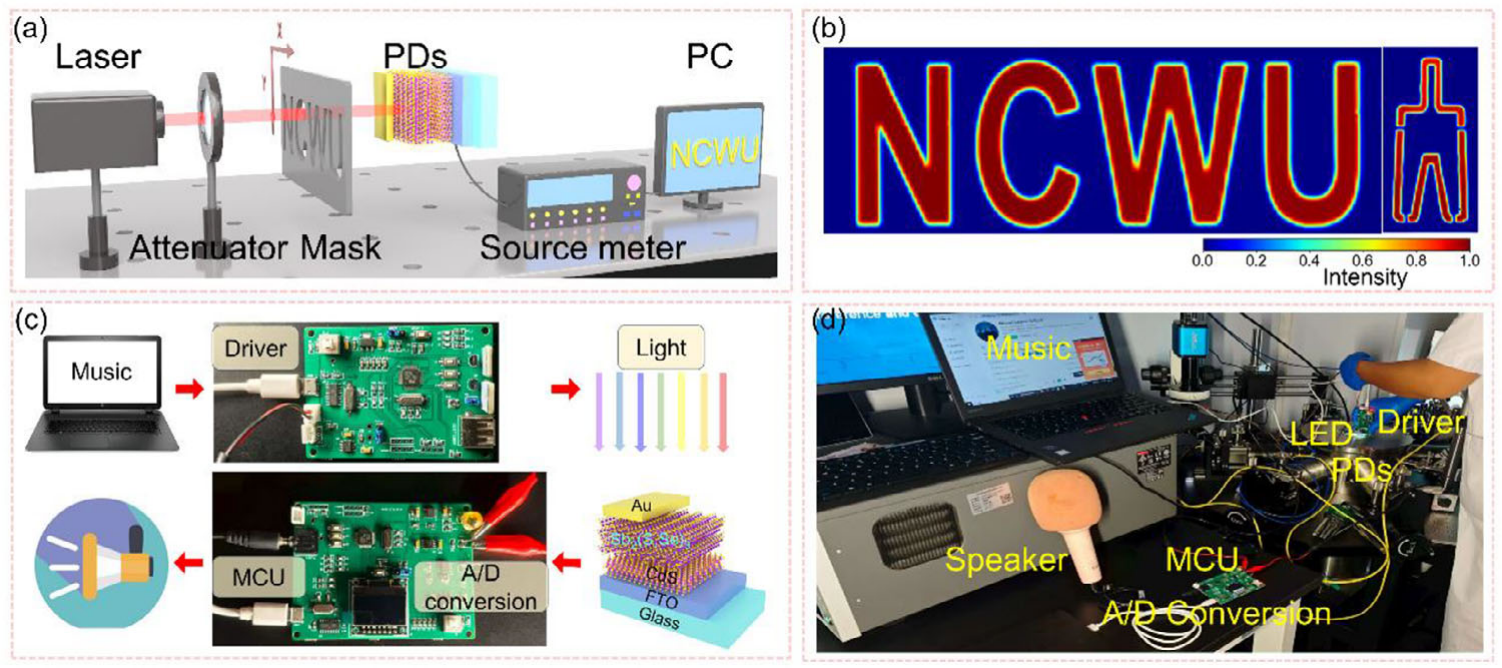
(a) Schematic illustration of the experimental setup for the image sensing application of the Sb_2_(S,Se)_3_/CdS heterojunction PDs. (b) The corresponding current mapping of ‘NCWU’ plough patterns logo and the color bar indicates the normalized current intensity (0–1.0). (c) Schematic diagram of the entire visible light system model. (d) The VLC test scenario diagram based on the Sb_2_(S,Se)_3_/CdS PD.

The excellent performance characteristics of the Sb_2_(S,Se)_3_/CdS heterojunction PDs enable their successful implementation as high‐sensitivity sensing pixels, security surveillance sensors, and digital photography detectors. Figure 6a illustrates the imaging measurement setup, where a photomask is mounted on a precisely controlled 2D translation stage and positioned between the light source and the PD [62]. During measurement, the laser beam passes through the pattern photomask and illuminates the PD, while a semiconductor parameter analyzer and a computer simultaneously record both the photocurrent signals and the corresponding spatial coordinates. This synchronized data acquisition enables the direct conversion of electrical signals into a 2D current map, where the contrast between dark current (background) and photocurrent (illuminated regions) forms the basis for image reconstruction. Figure 6b demonstrates the excellent imaging capability of the device under 785 nm illumination, which clearly resolves both the institutional abbreviation “NCWU” and a detailed plough symbol with remarkable clarity and edge definition. The reconstructed images show excellent shape fidelity and spatial consistency with the original photomask patterns, validating both the high resolution of the imaging system and the reliable pixel‐level performance of the Sb_2_(S,Se)_3_/CdS heterojunction PDs.

To further validate the communication capabilities of the Sb_2_(S,Se)_3_/CdS heterojunction PDs, we implemented them as optical receivers in a VLC system for real‐time voice transmission. Figure 6c presents the schematic diagram of the optical communication process. The audio signals were first converted into the intensity‐modulated visible light with a light‐emitting diode. Then the resulting optical signals are detected by the Sb_2_(S,Se)_3_/CdS heterojunction PD, whose photocurrent output is subsequently processed through amplification and filtering circuits before being reconstructed into audible sound via a speaker. The physical implementation of this VLC setup is shown in Figure 6d, confirming the seamless integration of the Sb_2_(S,Se)_3_/CdS heterojunction PD with visible‐light receiver circuitry. Experimental results demonstrate that the Sb_2_(S,Se)_3_/CdS heterojunction PD enables stable and high‐fidelity voice transmission, with the reproduced audio exhibiting smooth waveforms and minimal distortion (Supplementary Video S1). The successful implementation of this VLC audio system and its excellent structural stability against temperature fluctuations highlight the distinct advantages of Sb_2_(S,Se)_3_/CdS heterojunction PDs over conventional silicon‐based devices. Combined with the previously demonstrated broadband response, extreme temperature stability, and successful application in imaging and VLC, solution‑processed Sb_2_(S,Se)_3_ devices emerge as highly competitive candidates for next‑generation optoelectronic systems capable of operating in demanding environments.

## 3 Conclusion

In summary, we have successfully engineered a high‐quality Sb_2_(S,Se)_3_/CdS heterojunction PD via a feasible hydrothermal deposition method. The Sb_2_(S,Se)_3_‐based PDs achieve a high responsivity of 0.6 A W^−1^, an excellent specific detectivity of 7.68 × 10^12^ Jones, and a broad 3 dB bandwidth of 175 kHz, which establishes a new benchmark for solution‐processed, self‐powered PDs. More importantly, it exhibits a superior operational stability across an ultra‐broad temperature range from 10 to 575 K without encapsulation, which is a critical advantage for real‐world applications. The practical viability of our Sb_2_(S,Se)_3_/CdS heterojunction PDs is unequivocally demonstrated through their successful integration as a high‐speed receiver in a visible light communication system and as a sensitive pixel in high‐resolution imaging. This work not only provides a novel and robust material platform but also opens a practical route for the next generation of optoelectronic technologies, with promising prospects in medical near‐infrared imaging, aerospace extreme‐environment sensing, and security multispectral surveillance.

## Conflicts of Interest

The authors declare no conflicts of interest.

## Supporting information




**Supporting File**: advs74867‐sup‐0001‐SuppMat.docx.

**Supporting File**: advs74867‐sup‐0002‐VideoS1.mp4.

## Data Availability

The data that support the findings of this study are available from the corresponding author upon reasonable request.
