# Supplementary material for: Toward Next‐Generation High‐Speed Communication and Imaging: Solution‐Processed Sb2(S,Se)3/CdS Heterojunction for Ultrafast Self‐Powered Photodetector
Source: Adv Sci (Weinh). 2026 Mar 15;13(30):e23110. doi: 10.1002/advs.202523110 (PMC13248807; doi:10.1002/advs.202523110)
Supplement: Supplementary file 1 — Supporting File: advs74867‐sup‐0001‐SuppMat.docx. [file ADVS-13-e23110-s002.docx]

**Toward Next-Generation High-Speed Communication and Imaging: Solution-Processed Sb_2_(S,Se)_3_/CdS Heterojunction for Ultrafast Self-Powered Photodetector**

Xuhua Xiao, Yichao Wang, Jingxuan Bian, Hailong Wang, Yongqiang Wang^*^, Jinpeng Xu^*^, Junwei Chen^*^

X. Xiao

School of Electronic Engineering

North China University of Water Resources and Electric Power

Zhengzhou 450000, P. R. China

Y. Wang, J. Chen
School of Microelectronics

Hefei University of Technology

Hefei 230009, P. R. China

E-mail: [jwchen@hfut.edu.cn](mailto:jwchen@hfut.edu.cn)

J. Bian, H.Wang, J. Xu

Institute of Physics

Henan Academy of Sciences

Zhengzhou 450046, P. R. China

E-mail:[xujinpeng@hnas.ac.cn](mailto:xujinpeng@hnas.ac.cn)

Yongqiang Wang

The Affiliated Cancer Hospital of Zhengzhou University

Henan Cancer Hospital

Zhengzhou 450008, P. R. China

E-mail:wyq331@mail.ustc.edu.cn

**Experimental Section**

**CdS Film Fabrication.** The device structure was FTO/CdS/Sb_2_(S,Se)_3_/Au. The FTO-coated conductive glass was ultrasonically cleaned sequentially with detergent, acetone, isopropanol, and deionized water (DI), for 15 minutes in each solvent. Substrates were subjected to UV-ozone cleaning prior to CdS layer deposition.

The CdS thin films were deposited via a conventional chemical bath deposition (CBD) method. Briefly, 22.5 mL of 15 mM cadmium sulfate (CdSO_4_) aqueous solution, 30 mL of ammonia water (NH_3_·H_2_O), and 165 mL of deionized (DI) water were first mixed and magnetically stirred for 2 minutes.^[1]^ Subsequently, 11.25 mL of 1.5 M thiourea (TU) solution was added dropwise into the aforementioned mixture under continuous magnetic stirring. The CBD process was carried out at a constant temperature of 66 °C for 13.5 minutes. After deposition, the as-deposited CdS layer was immersed in a cadmium chloride (CdCl_2_) solution (20 mg·mL^−1^ in absolute methanol), followed by a post-deposition heat treatment at 400 °C for 10 minutes in ambient air. Finally, the substrates were allowed to cool down to room temperature naturally.

**Sb_2_(S,Se)_3_ Film Fabrication.** The Sb_2_(S,Se)_3_ layer was then fabricated by a typical hydrothermal synthesis. ^[2–4]^ Precisely, the precursor solution was prepared by dissolving 20 mM K_2_Sb_2_(C_4_H_2_O_6_)·3H_2_O (APT, 99.99%, Macklin), 160 mM Na_2_S_2_O_3_·5H_2_O (STS, 99.0%, Sinopharm), and 5 mL Na_2_SeSO_3_ (SSS) solution in 40 mL of DI water. The solution was stirred at room temperature for 1 minute and then transferred to a Teflon-lined autoclave. FTO/CdS substrates (face-down, glued to a glass strip using double-sided adhesive) were fixed in the reactor. The deposition was conducted at 135 °C for 150 minutes. After cooling, the films were rinsed with DI water and absolute ethanol, air-dried, and annealed in a tube furnace at 360 °C for 10 minutes. Au (~80 nm thick) was thermally evaporated onto the Sb_2_(S,Se)_3_ films as the top electrode under 5.0×10^−4^ Pa, using a mask to define an effective active area of 2.25 mm^2^.

**Film and Device Performance Characterizations:** The SEM images were acquired using a JSM-IT800. The UV-vis-NIR absorption, XRD and other measurements were performed as described in our previous work. ^[3–5]^ Raman and PL spectra were collected using a confocal micro-Raman system (Alpha300R, WITec) excited by a 532 nm laser. All electrical/optoelectrical measurements were carried out by a semiconductor parameter analyzer (Keithley 4200A-SCS, Tektronix). The device was connected in series with a 50 Ω resistance in 3 dB Bandwidth Measurement with a785 nm. Measurements at 0-300 K were conducted in a low-temperature probe station under a vacuum level of ~8×10^−4^ Pa, where the temperature was precisely regulated using a temperature controller (Model 336, Lake Shore). Measurements above 300 K were conducted in ambient air using a hot stage. The light sources were lasers with wavelengths of 375 ~ 1064 nm.


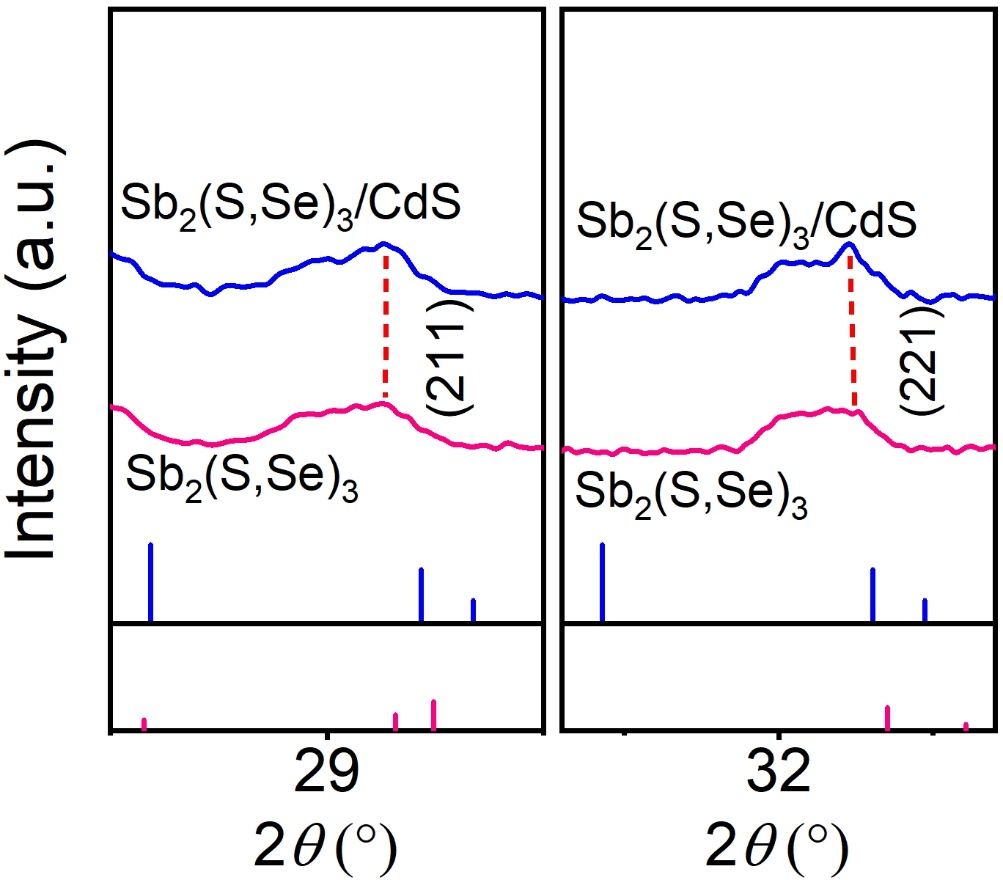


**Figure S1.** The magnified XRD patterns for the (211) and (221) crystal plane the Sb_2_(S,Se)_3_ and Sb_2_(S,Se)_3_/CdS films.

Notes to **Figure S1:** A significant enhancement in the intensity of the (211) and (221) peaks are observed in the Sb_2_(S,Se)_3_/CdS film after treatment, compared to the untreated the reference Sb_2_(S,Se)_3_ sample, which is indicative of improved crystallinity.


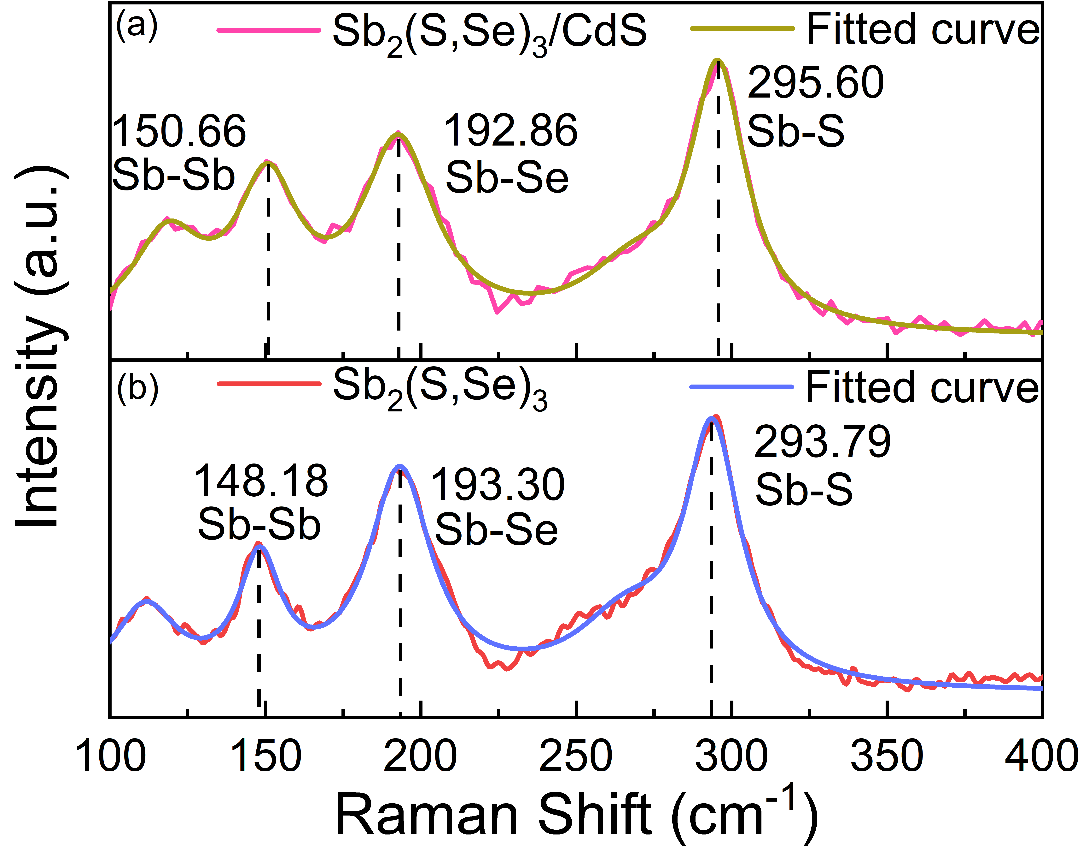


**Figure S2.** Raman spectra of Sb_2_(S,Se)_3_ and Sb_2_(S,Se)_3_/CdS heterojunction films.

**Table S1.** The parameter of Raman spectra for Sb_2_(S,Se)_3_ and Sb_2_(S,Se)_3_/CdS films.

| Sample | Peak | Position (cm^−1^) | FWHM (cm^−1^) | Assignment |
| --- | --- | --- | --- | --- |
| Sb_2_(S,Se)_3_ | Peak 1 | 193.30 | 24.80 | Sb-Se |
|  | Peak 2 | 293.79 | 21.96 | Sb-S |
| Sb_2_(S,Se)_3_/CdS | Peak 1 | 192.86 | 24.22 | Sb-Se |
|  | Peak 2 | 295.61 | 21.81 | Sb-S |


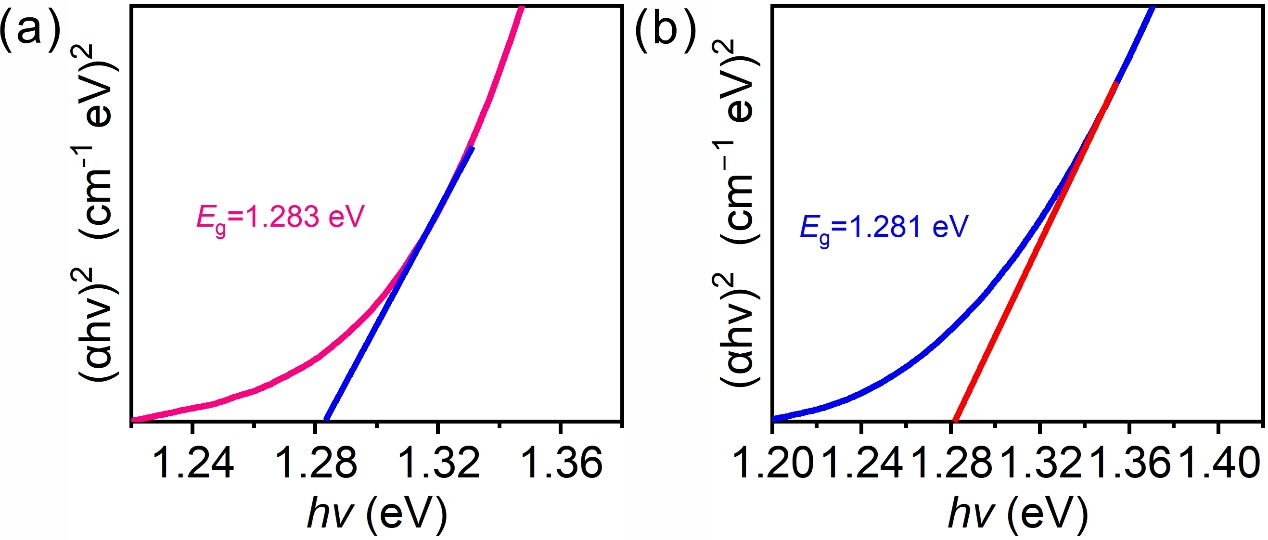


**Figure S3.** Absorption spectra and *E*_g_ of (a) Sb_2_(S,Se)_3_ and (b) Sb_2_(S,Se)_3_/CdS films.


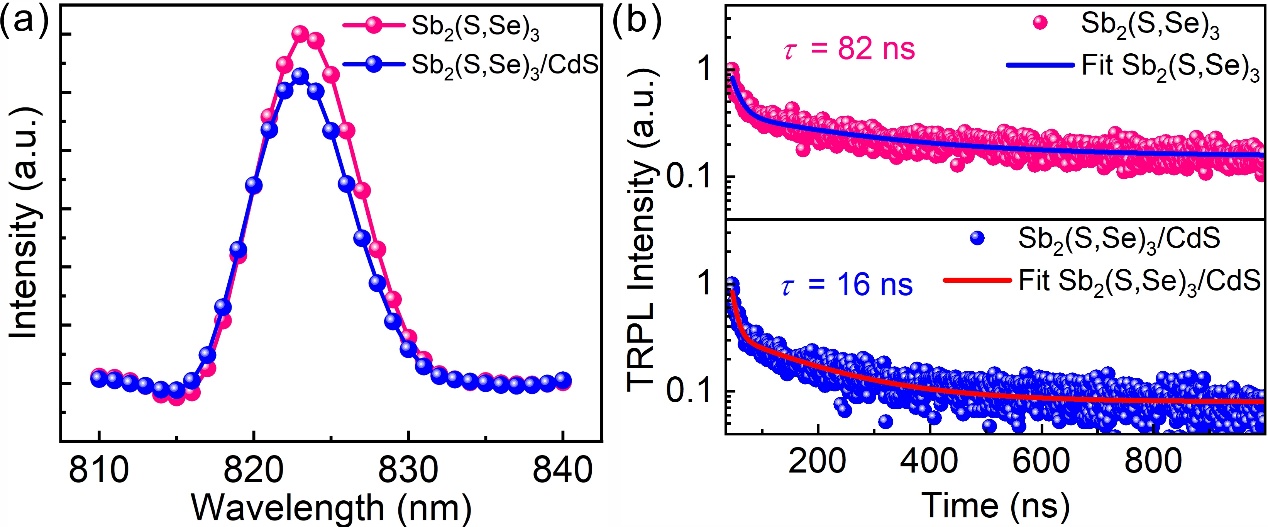


**Figure S4.** (a) PL spectra of the Sb_2_(S,Se)_3_ and Sb_2_(S,Se)_3_/CdS films. (b) TRPL decay and fitting curve of the Sb_2_(S,Se)_3_ and Sb_2_(S,Se)_3_/CdS films.

Notes to·**Figure S4**: The TRPL spectra are fitted using a biexponential model^[6,7]^, and the fitted curves (solid lines) are in good agreement with the experimental data (scattered points). Obviously, compared with single Sb_2_(S,Se)_3_ film, the construction of Sb_2_(S,Se)_3_/CdS heterojunction improves interfacial charge transfers and separation, which will benefit the photoelectric performance of the devices.^[8]^

**Table S2.** The fitting parameter of TRPL

| Parameters | Sb_2_(S,Se)_3_ | Sb_2_(S,Se)_3_/CdS |
| --- | --- | --- |
| *τ*_1_ | 14.93 ns | 8.82 ns |
| *τ*_2_ | 244.37 ns | 156.34 ns |
| *τ*_ave._ | 82 ns | 16 ns |
| A_1_ | 10.64 | 106.95 |
| A_2_ | 0.27 | 0.33 |

Notes to **Table S2**: The TRPL spectra are fitted using a biexponential model, in which τ_1_ is associated with rapid recombination processes, typically arising from surface or interface defects and τ_2_ corresponds to slower bulk-phase recombination. The average carrier lifetime (τ_avg._) is calculated as a weighted average of τ_1_ and τ_2_, reflecting the fractional contributions of each recombination pathway. For the Sb_2_(S,Se)_3_/CdS heterojunction, the *τ*_1_ is a typical characteristic of efficient photocarrier separation at the heterojunction interface, thus avoiding the recombination loss of photocarriers in the bulk phase. Meanwhile, the reduced *τ*_2_ also indicates the suppressed bulk non-radiative recombination, and the increased proportion of the fast decay component further confirms the promoted interfacial carrier transport dynamics. Collectively, these TRPL results fully demonstrate that the Sb_2_(S,Se)_3_/CdS heterojunction possesses more excellent photoelectric conversion and carrier transport properties than the pure Sb_2_(S,Se)_3_ film.

| **Table S3.** The atomic ratio in XPS results of Sb_2_(S,Se)_3_ and Sb_2_(S,Se)_3_/CdS films. | | |
| --- | --- | --- |
| Elemental | Sb_2_(S,Se)_3_/CdS | Sb_2_(S,Se)_3_ |
|  | Atomic (%) | Atomic (%) |
| S | 53.21 | 55.90 |
| Se | 15.60 | 13.45 |
| Sb | 31.19 | 30.65 |

Notes to **Table S3**: XPS analysis reveals a shift in surface elemental composition: the Sb_2_(S,Se)_3_ reference film exhibits a composition of Sb_2_(S_0.81_Se_0.19_)_3_, whereas the film deposited on CdS layer shows an increased Se content, corresponding to Sb_2_(S_0.77_Se_0.23_)_3_.





**Figure S5.** Noise current spectral density (S(f)) of the Sb_2_(S,Se)_3_/CdS heterojunction photodetector (zero bias).

Notes to **Figure S5**: At a frequency of <100 Hz, the noise is mainly contributed by flicker 1/f noise that produced by scattering at Sb_2_(S,Se)_3_/CdS thin film and at the device interface. At a higher frequency, the noise is primarily decided from thermal noise that generated by random thermal motion of charge carriers. Noise equivalent power (NEP) and total noise determined realistic detectivity (*D^*^*) were calculated using the following equation^[9]^: NEP=(*i*_noise_^2^)^1/2^/*R*; *D*^*^=(*AΔf*)^1/2^ /NEP; where *R* is the responsivity, *i*_n_ is noise current density. *i*_n_, *NEP* are calculated to be 2.914 × 10^−12^ A Hz^−1/2^, 5.02 × 10^−12^ W Hz^−1/2^ based on frequency-dependent noise power spectral density measurements.





**Figure S6.** External quantum efficiency (EQE) of the Sb_2_(S,Se)_3_/CdS self-powered photodetector versus incident light intensity (zero bias).

Notes to **Figure S6**: *EQE = hcR/(λq)*, where h is Planck’s constant, *c* is the speed of light, *R* is the responsivity of the Sb_2_(S,Se)_3_/CdS PDs, *λ* is the wavelength of incident light, and *q* is the electron charge.





**Figure S7**. (a) Dependence of photocurrent on incident light intensity for the ref-Sb_2_(S,Se)_3_ film at 0 V bias. (b) Responsivity and specific detectivity of the ref-Sb_2_(S,Se)_3_ film versus incident light intensity.





**Figure S8.** Optoelectronic characterization tested at 375 nm, 450 nm, 532 nm, 1064 nm. (a-d) The current-voltage characteristic curves. (e-h) Photocurrent response tested at 0 V bias. (i-k) *R*, *D^*^* and *EQE* of the photodetector as a function of light intensity (l) Dependence of photocurrent on the intensity of light power at 0 V bias.





**Figure S9.** (a) Schematic of the fast response measurement setup for the Sb_2_(S,Se)_3_/CdS heterojunction PDs. Responses of the device to pulsed light signals with frequencies of (b) 2 Hz, (c) 22 kHz, and (d) 200 kHz.


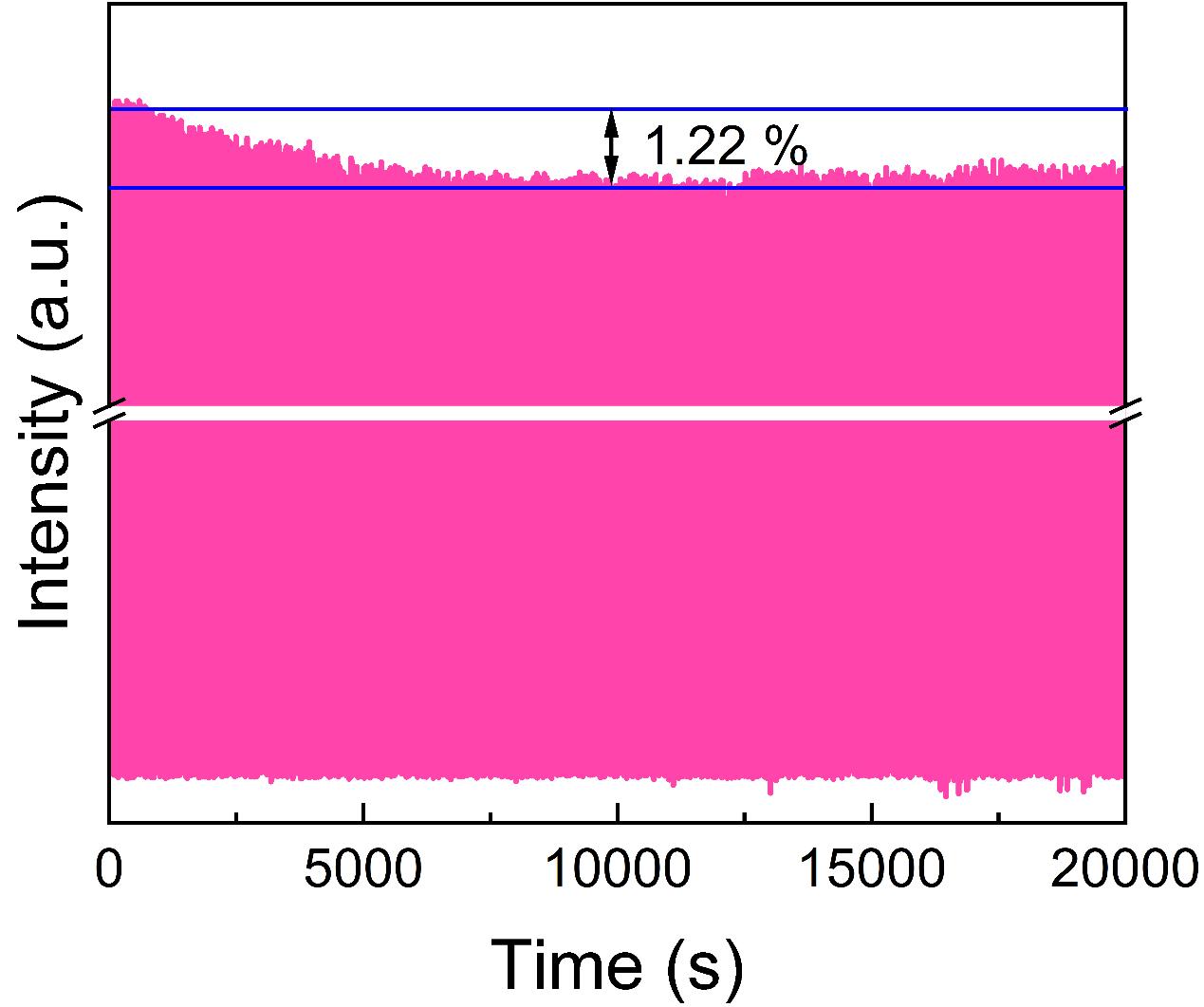


**Figure S10.** The performance degradation behavior of Sb_2_(S,Se)_3_/CdS PDs without encapsulation after 20,000 consecutive working cycles.


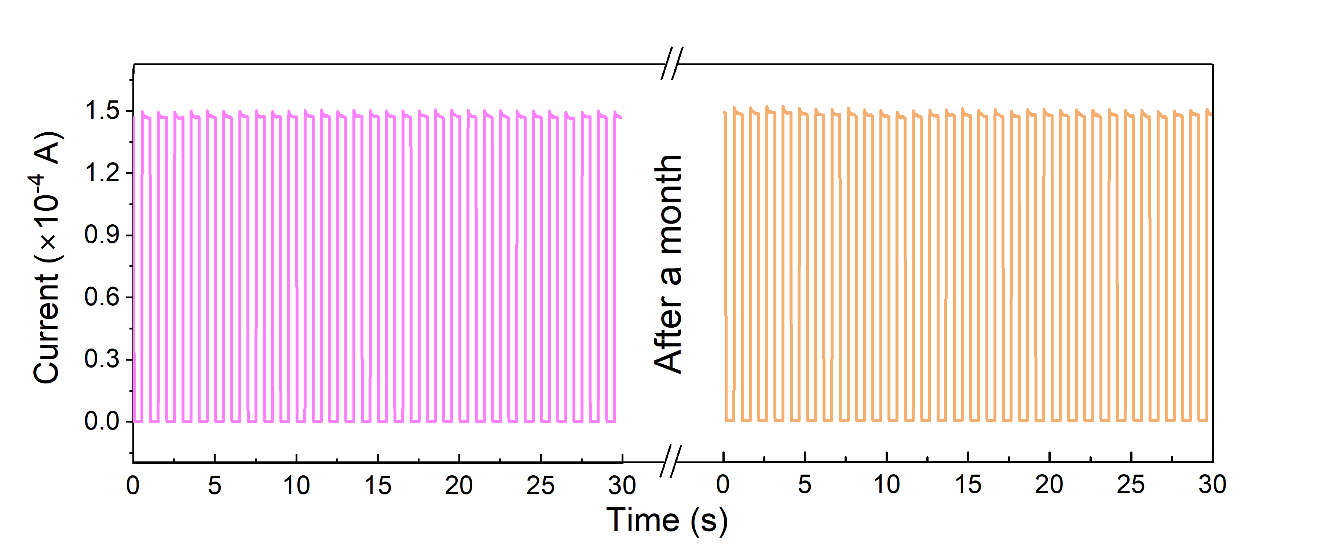


**Figure S11.** Storage stability of the unencapsulated Sb_2_(S,Se)_3_/CdS heterojunction: photoresponse performance after storage for 1 month in a glovebox.





**Figure S12.** Optoelectronic performance of the Sb_2_(S,Se)_3_/CdS heterojunction photodetector under 532 nm illumination at 10 K, 0 V bias. (a) The current-voltage characteristic curves of at 10 K. (b) Photocurrent response tested at 0 V bias. (c) *R*, *D^*^* versus light intensity.


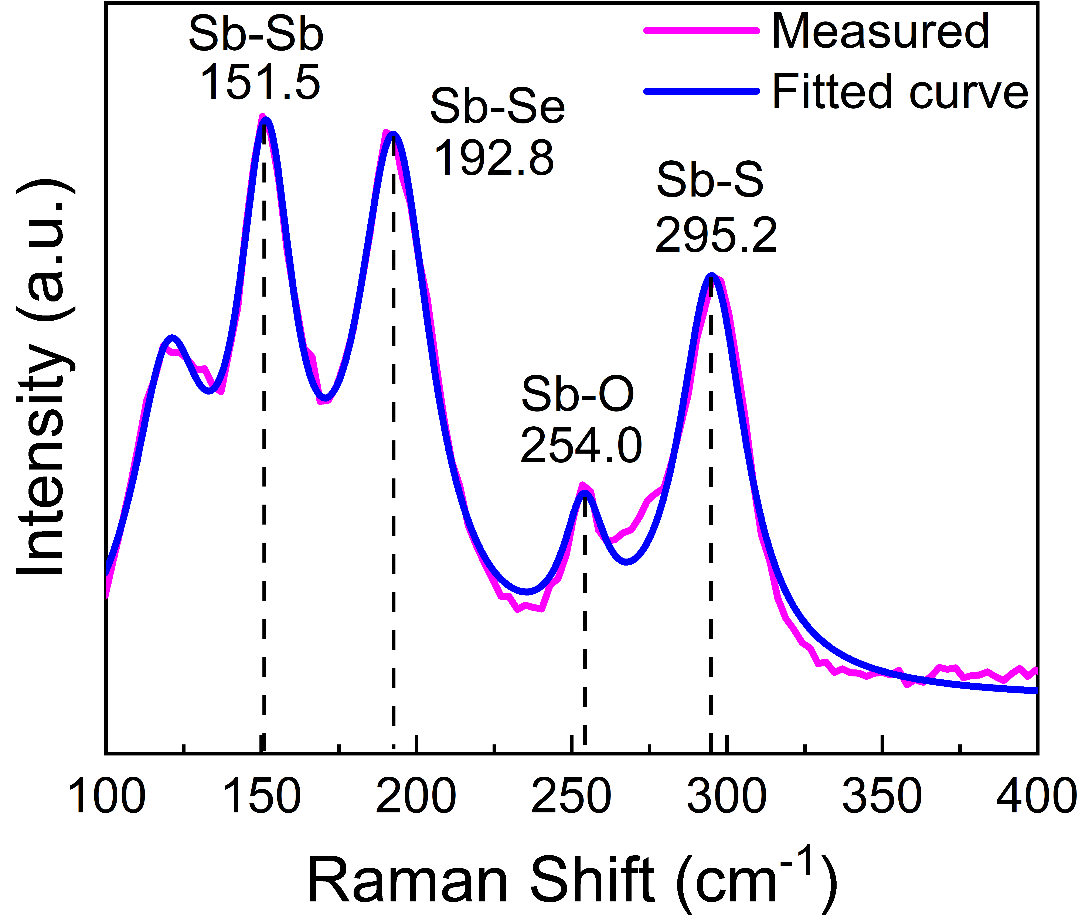


**Figure S13.** Raman spectra of Sb_2_(S,Se)_3_/CdS heterojunction after temperature tests.

**Reference**

[1] X. Peng, Z. Ma, Z. He, R. Tang, J. Li, S. Sheng, T. Wu, Y. Hu, Z. Cai, Z. Jiang, Y. Li, C. Zhu, R. Cao, X. Zheng, T. Chen, *Adv. Funct. Mater.* **2025**, *35, 2503314*.

[2] J. Chen, G. Li, C. Gao, S. Sheng, C. Ruan, Y. Wang, Z. Xu, R. Tang, C. Chen, Y. Zhang, T. Chen, J. Xu, *Adv. Funct. Mater.* **2026**,*36*, e12587.

[3] J. Chen, C. Xu, G. Li, Z. Xu, Y. Wang, Y. Zhang, C. Chen, M. Wang, L. He, J. Xu, *Angew. Chem. Int. Ed.* **2024**, *63*, e202409609.

[4] L. Zhu, R. Liu, Z. Wan, W. Cao, C. Dong, Y. Wang, C. Chen, J. Chen, F. Naveed, J. Kuang, L. Lei, L. Cheng, M. Wang, *Angew. Chem. Int. Ed.* **2023**, *62*, e202312951.

[5] Y. Wang, D. Yang, M. Jin, Z. Wan, W. Cao, F. Naveed, J. Kuang, C. Zheng, C. Wang, J. Chen, Y. Dong, M. Wang, C. Chen, *Adv. Energy Mater.* **2025**, *15*, 2502805.

[6] X. Wang, J. Bhosale, J. Moore, R. Kapadia, P. Bermel, A. Javey, M. Lundstrom, *IEEE J. Photovolt.* **2015**, *5*, 282.

[7] P. M. Jundt, D. Kuciauskas, J. R. Sites, *IEEE J. Photovolt.* **2022**, *12*, 501.

[8] X. Liang, X. Wang, Q. Chang, B. Yang, W. Dang, Z. Zhang, Y. Guo, L. Yang, Z. Li, *Energy Environ. Sci.* **2024**, *17*, 9499.

[9] J. Xu, X. Luo, X. Lin, X. Zhang, F. Liu, Y. Yan, S. Hu, M. Zhang, N. Han, X. Gan, Y. Cheng, W. Huang, *Adv. Funct. Mater.* **2024**, *34, 2310811*.
